# Effect of Bcl-2 rs956572 Polymorphism on Age-Related Gray Matter Volume Changes

**DOI:** 10.1371/journal.pone.0056663

**Published:** 2013-02-20

**Authors:** Mu-En Liu, Chu-Chung Huang, Albert C. Yang, Pei-Chi Tu, Heng-Liang Yeh, Chen-Jee Hong, Jin-Fan Chen, Ying-Jay Liou, Ching-Po Lin, Shih-Jen Tsai

**Affiliations:** 1 Department of Psychiatry, Kaohsiung Veterans General Hospital, Kaohsiung, Taiwan; 2 Department of Biomedical Imaging and Radiological Sciences, National Yang-Ming University, Taipei, Taiwan; 3 Department of Psychiatry, Taipei Veterans General Hospital, Taipei, Taiwan; 4 School of Medicine, National Yang-Ming University, Taipei, Taiwan; 5 Center for Dynamical Biomarkers and Translational Medicine, National Central University, Chungli, Taiwan; 6 Department of Medical Research and Education, Taipei Veterans General Hospital, Taipei, Taiwan; 7 Taipei Veterans Home, New-Taipei City, Taiwan; 8 Department of Pathology, Tao-Yuan Veterans Hospital, Tao-Yuan County, Taiwan; 9 Institute of Neuroscience, National Yang-Ming University, Taipei, Taiwan; University of California San Francisco, United States of America

## Abstract

The anti-apoptotic protein B-cell CLL/lymphoma 2 (Bcl-2) gene is a major regulator of neural plasticity and cellular resilience. Recently, the Bcl-2 rs956572 single nucleotide polymorphism was proposed to be a functional allelic variant that modulates cellular vulnerability to apoptosis. Our cross-sectional study investigated the genetic effect of this Bcl-2 polymorphism on age-related decreases in gray matter (GM) volume across the adult lifespan. Our sample comprised 330 healthy volunteers (191 male, 139 female) with a mean age of 56.2±22.0 years (range: 21–92). Magnetic resonance imaging and genotyping of the Bcl-2 rs956572 were performed for each participant. The differences in regional GM volumes between G homozygotes and A-allele carriers were tested using optimized voxel-based morphometry. The association between the Bcl-2 rs956572 polymorphism and age was a predictor of regional GM volumes in the right cerebellum, bilateral lingual gyrus, right middle temporal gyrus, and right parahippocampal gyrus. We found that the volume of these five regions decreased with increasing age (all P<.001). Moreover, the downward slope was steeper among the Bcl-2 rs956572 A-allele carriers than in the G-homozygous participants. Our data provide convergent evidence for the genetic effect of the Bcl-2 functional allelic variant in brain aging. The rs956572 G-allele, which is associated with significantly higher Bcl-2 protein expression and diminished cellular sensitivity to stress-induced apoptosis, conferred a protective effect against age-related changes in brain GM volume, particularly in the cerebellum.

## Introduction

Aging strongly affects brain morphology, which may contribute to cognitive change over time [1], [2]. Good et al. [1] reported that aging predominantly and substantially affects gray matter (GM), and that GM volume decreased linearly with age. Others have reported that several of the age-associated changes in brain volume are probably nonlinear. Sullivan and Pfefferbaum [3] found that, during the normal aging process, initial growth in the cortical GM compartment occurred until the age of 5, followed by a steady decline in volume throughout the remaining lifespan. In a 5-year MRI follow-up study, however, Van Haren et al. [4] assessed 113 participants, and observed essentially no decrease until the age of 30 years. From that age onward, cerebral volume gradually decreased. Furthermore, studies of healthy volunteers reported significant trends in age-related volume reduction in certain regions of the brain, including the hippocampus [5], the cerebellum [1], and the prefrontal [2], temporal [2], and occipital lobes [5].

Twin studies have shown that many aspects of brain structure are highly heritable, with heritability estimates ranging from 82% for gray matter to 88% for white matter [6], [7]. A longitudinal study of 71 twin pairs by Prefferbaum et al. [11] showed that genetic contributions to variability in brain structure were high at baseline and at a 4-year follow-up. Although the genetic components of age-related changes in the human brain volume remain largely unknown, several candidate genes have been suggested to influence age-related changes in brain structure. Sublette et al. [8] reported that an allelic variant of brain-derived neurotrophic factor (BDNF) was associated with age-related changes in the amygdala volume, and Nemoto et al. [9] reported that the same BDNF allelic variant influenced age-related changes in brain morphology. The apolipoprotein E genotype has also been shown to have an impact on age-related GM volume loss [10]. The findings of these studies suggest that genetic variation may influence age-related changes in brain morphology.

The anti-apoptotic protein B-cell CLL/lymphoma 2 (Bcl-2) is a major inhibitor of apoptotic and necrotic cell death [12]. Bcl-2 also plays critical roles in neuronal morphogenesis and synaptic plasticity [13], [14], and altered Bcl-2 function has been proposed to contribute to the impairment of cellular plasticity and resilience in neuropsychiatric patients [12]. Bcl-2 may support central neurons through intracellular calcium signaling, which stimulates the regenerative response and neuronal differentiation [15], and this mechanism may influence aging processes and pathogenesis in neurodegenerative disease [16]. These findings collectively suggest that Bcl-2 may play a critical role in the modulation of aging processes in the brain [17], [18].

Uemura et al. [19] recently demonstrated that the intronic single nucleotide polymorphism (SNP) Bcl-2 rs956572 influences Bcl-2 function in B lymphoblast cell lines derived from bipolar disorder patients. The levels of Bcl-2 mRNA and protein were lowest in cell lines of patients with the G/G genotype, compared to that of patients with the other functional genotypes, G/A and A/A. In contrast, an earlier study using similar cell lines found that the A/A genotype was associated with significantly lower Bcl-2 expression and greater cellular sensitivity to stress-induced apoptosis, compared with the G/G genotype [20]. However, both studies showed that the Bcl-2 polymorphism was associated with intracellular calcium homeostasis in lymphoblast cells derived from bipolar disorder patients.

A growing body of evidence indicates that a relationship exists between altered Bcl-2 expression and the neurodegenerative process [18], and that calcium signaling is responsible for neuronal aging and degeneration [21]. Increased vulnerability to Bcl-2-related apoptosis induced by physiological stressors has been suggested to contribute to the reductions in regional cerebral volumes, neurons, and glial cells in patients with mood disorders [22]. An investigation of the genetic effect of the Bcl-2 rs956572 polymorphism on regional brain-GM volumes in adults aged 19 to 60 years using optimized voxel-based morphometry (VBM) showed that G homozygotes displayed larger GM volumes in the left ventral striatum, compared with that of the A-allele carriers [23]. Recently, we investigated the genetic effects of Bcl-2 rs956572 on regional GM volumes in elderly men [24]. We found that G homozygotes had significantly larger GM volumes in the left precuneus, the right lingual gyrus, and the left superior occipital gyrus, compared with those of the A-allele carriers. Previous studies have reported age-related influences on Bcl-2 expression in specific brain regions [25], [26]. Thus, increased vulnerability to Bcl-2-related apoptosis may be involved in the aging process [27]. Considering these findings and the role that Bcl-2 plays in neural plasticity and cellular resilience, we hypothesized that the Bcl-2 rs956572 allelic variant may contribute to age-related changes in GM volumes. Therefore, we evaluated the relationship between the Bcl-2 rs956572 genotype and age-related changes in regional GM volumes based on the results of optimized VBM over a broad age range.

## Materials and Methods

### 2.1 Participants and Instruments

We recruited 330 healthy participants in northern Taiwan (mean age: 56.2±22.0 years, range: 21–92; 57.9% males). Each participant was evaluated by a trained research assistant using the Mini-International Neuropsychiatric Interview [28]. The participants were screened using the Mini-Mental Status Examination (MMSE) and the Clinical Dementia Rating Scale. The exclusion criteria included the following: (1) Any Axis-I diagnosis according to the DSM-IV, such as mood disorders or psychotic disorders; (2) neurological disorders, such as dementia, head injury, stroke, or Parkinson disease; (3) illiteracy; (4) participants with an MMSE score below 24; (5) any chronic illness under medical control, including malignancy, heart failure, lung disease, and diabetes; and (6) a Clinical Dementia Rating Scale score over 0.5 for participants aged 65 and over.

The cognitive functioning of the participants was evaluated using the MMSE and the Wechsler Digit Span Task tests. All participants had sufficient visual and auditory acuity to undergo cognitive testing. The 30-point MMSE cognitive test was designed for screening cognitive impairment in cross-cultural studies. Our research was conducted in accordance with the Declaration of Helsinki, and was approved by the Institutional Review Board of Taipei Veterans General Hospital. Written, informed consent was obtained from all the participants.

### 2.2 Genotyping

Genomic DNA was extracted from peripheral blood with a commercial kit (Qiagen, Gentra Puregene Blood Kit). Genotyping procedures for identifying the rs956572 was performed by the polymerase chain reaction (PCR)-restriction fragment length polymorphism method. The following PCR primers, which were synthesized by MISSION BIOTECH Co. (Taiwan) were used in the present study: forward, 5- AGAGGAAAGAGCACACAC-3 and reverse, 5- AGAACTCTACTTCCAGGC-3. PCR reactions were performed in a 12.5 ul final volume containing 1×PCR buffer, 1.0 mM Mg2+, 0.2 mM dNTPs, 5 pmol of each primer and 0.3 U Taq polymerase. PCR cycles were the following: 95°C for 5 min followed by 35 cycles each of 95°C for 30 s, 53°C for 30 s, 72°C for 30 s. A final extension step was undertaken at 72°C for 5 min. The 567 base pair sequences of the Bcl-2 gene were amplified by PCR, and their products were digested with restriction endonuclease Ddel (New England BioLabs Inc.). The ancestral allele G yielded three bands of 298, 108 and 161 bp while the mutant allele A yielded two bands of 406 and 161 bp.

### 2.3 MRI Acquisition

All MR scanning was performed at National Yang-Ming University, Taiwan, using a 3.0 T Siemens MRI scanner with 12 channel head coil (Siemens Magnetom Tim Trio, Erlangen, Germany). High-resolution structural MR images were acquired with 3D magnetization prepared rapid gradient echo sequence (TR = 2,530 ms, TE = 3.5 ms, TI = 1,100 ms, FOV = 256 mm, flip angle = 7°, matrix size = 256×256, 192 sagittal slices, voxel size = 1.0×1.0×1.0 mm, no gap). All the images were acquired parallel to the anterior commissure–posterior commissure line. To minimize motion artifact generated during image acquisition, each subject’s head was immobilized with cushions inside the coil. Each image was carefully checked by an experienced radiologist to ensure that they had no scanner artifacts, motion problems, or gross anatomical abnormalities.

### 2.4 DARTEL-based T1 VBM Analysis

Individual T1-weighted volumetric images were processed using Gaser’s VBM8 toolbox (http://dbm.neuro.uni-jena.de) within Statistical Parametric Mapping (SPM8, Wellcome Institute of Neurology, University College London, UK) executed in MATLAB 2010a (The MathWorks, Natick, MA, USA) under Linux 64-bit environment with recommended settings. VBM processing was performed as following procedure: 1) the anterior commissure was set as the origin of each T1-weighted image. 2) Segmentation approach in the VBM8 toolbox was applied in the initial native space that combined the nonlocal means denoising filter [29] and adaptive maximum a posteriori segmentation approach [30] with partial volume estimation technique [31]. Images were further refined by applying an iterative hidden Markov random field model [32] to remove isolated voxels which were unlikely to belong to a determinate tissue type, and to improve the quality of tissue segmentation. 3) To achieve higher accuracy of registration between subjects, the native space GM, white matter (WM), and CSF segments were initially affine registered to the tissue probability maps in the Montreal Neurological Institute (MNI) standard space (http://www.mni.mcgill.ca/). 4) All affine registerted tissue segments were iteratively registered to group-based templates, which were generated from all images included in the current study through nonlinear warping using DARTEL (Diffeomorphic Anatomical Registration Through Exponentiated Lie Algebra) toolbox [33] that implemented in SPM8. 5) The non-linear deformation parameters obtained in the previous step were used to modulate the GM, WM, and CSF tissue maps of participants’ brains so as to compare actual volumetric differences across groups. 6) Finally, the modulated tissue segments were converted into an isotropic voxel resolution of 1×1×1 mm^3^. All normalized, segmented, and modulated MNI standard space images were smoothed with an 8-mm Gaussian kernel ahead of tissue volume calculation and voxel-wised group comparisons. Segmented tissue volumes (i.e., GM, WM, and CSF) were estimated in cubic millimeters by counting the voxels representing GM, WM, and CSF in standard space. Total intracranial volume (TIV) was determined as the sum of GM, WM, and CSF volumes.

### 2.5 Statistical Analysis

Statistical analyses were performed using the SPSS 18.0 program (SPSS Inc., Chicago, IL). Student’s t-test and Chi-square test were applied to compare the continuous and categorical variables between the two groups (A-carriers, and G homozygotes), respectively. Smoothed modulated gray matter segments were analyzed with SPM8 utilizing the framework of General Linear Model (GLM). To investigate whether Bcl-2 SNP exhibiting age-related linear interaction to alter regional GMV between two genotypic groups, voxel-wised covariate interaction analysis was employed using Bcl-2 genotype as a condition and age as covariates, controlling sex and education level as nuisance variables. This analysis tested for any regional GMV showing genotype-by-age interactions. To avoid possible partial volume effects around the margin between GM and WM, all voxels with a GM probability value lower than 0.2 (range from 0 to 1) were eliminated. The statistical criteria of interaction analysis were deemed to be significant at threshold of uncorrected p-value <0.001 as well as extended cluster size more than 50 contiguous voxels. We used the icbm2tal function from the GingerALE toolbox (The BrainMap Development Team; http://brainmap.org/ale/index.html) to transform MNI coordinates into Talairach coordinates, and to minimize coordinate transformation discrepancy between MNI and Talairach space. Anatomical structures of the coordinates representing significant clusters were identified on the basis of the Talairach and Tournoux atlas [34]. All regional GMV were extracted and summed up from the peak coordinates showing significant differences.

## Results

**Figure 1:**
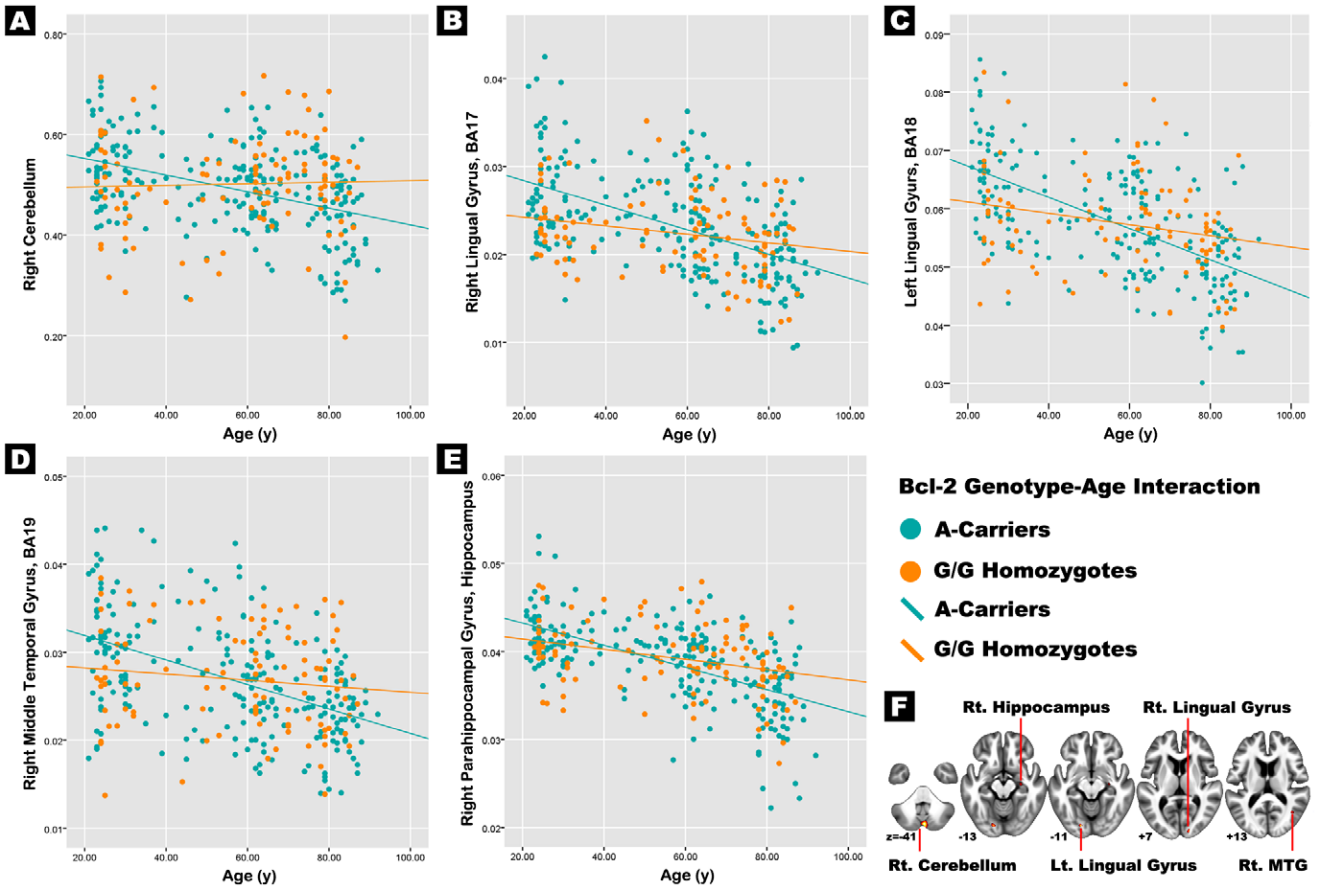
Interaction of *Bcl-2* genotype and age on regional gray matter volume. Interaction of Bcl-2 genotype and age on (**A**) right cerebellum, (**B**) right lingual gyrus (BA17), (**C**) left lingual gyrus (BA18), (**D**) right middle temporal gyrus (BA19), and (**E**) right parahippocampal gyrus (hippocampus). (**F**) Showing the interaction results of voxel-wised covariate analysis using Bcl-2 genotype as a condition and age as covariates, controlling sex and education level as nuisance variables (uncorrected p<0.001, cluster size larger than 50). Abbreviations: MTG, middle temporal gyrus; BA, Brodmann Area.

Of the 330 participants, 102 were G homozygotes, 65 had the A/A genotype, and 163 had the A/G genotype. There were no significant differences between the demographic and neuropsychological characteristics of the Bcl-2 G homozygotes and the A-allele carriers (Table 1). For GM volume, the Bcl-2 genotype was significantly associated with age-related changes in several brain regions. The association of the Bcl-2 rs956572 polymorphism with age was a predictor of regional GM volumes in the right cerebellum [F(1,328) = 13.77; P<.0001], the right lingual gyrus [BA17; F(1,328) = 11.6; P = .001], the left lingual gyrus [BA18; F(1,328) = 13.99; P<.0001], the right middle temporal gyrus [BA19; F(1,328) = 32.36; P = .001], and the right parahippocampal gyrus [hippocampus; F(1,328) = 11.06; P = .001], and this effect was most significant in the cerebellum for large voxel size (Table 2, Figure 1). Correlation analysis showed that the GM volume of these five areas significantly decreased with increasing age in the Bcl-2-A-allele carriers. No significant age-related changes in regional GM volume occurred in the G homozygotes. (Table 2, Figure 1).

**Table 1:** Demographical characteristics and preclinical assessments between *Bcl-2* genotype groups.

Demographic variables	A-Carriers	G/G	*P* value
	(n = 228)	(n = 102)	
Age (y)	55.9 (22.5)	57.0 (21.1)	.689
Sex (male/female)	135/93	56/46	.472
Education (y)	12.5 (6.1)	12.3 (6.7)	.771
Handedness (left/right)	6/222	4/98	.506
GMV (L)	0.78 (0.08)	0.78 (0.07)	.915
MMSE	27.9 (2.37)	27.7 (2.25)	.414
Digits Span Forward	13.4 (2.64)	13.8 (2.54)	.322
Digits Span Backward	7.68 (3.93)	7.07 (4.33)	.208

The variables are demonstrated as means (± standard deviation). Abbreviation: GMV, gray matter volume; MMSE, Mini-Mental Status Examination.

**Table 2:** Interaction of *Bcl-2* genotype and age on regional gray matter volume.

MNICoordinates			Voxel size	Anatomical Region		Brodmann Area	Main Effects	F-value	*P* value	Correlation (*r*)	
x	y	z								A-Carrier	G/G
							Bcl-2	10.32	.001		
2	−78	−41	868	Right	Cerebellum	−	Age	2.83	.094	−0.22*	−0.03
							Bcl-2× Age	13.77	<.0001		
							Bcl-2	14.21	<.0001		
16	−89	7	67	Right	Lingual Gyrus	Brodmann area 17	Age	11.37	.001	−0.29*	−0.09
							Bcl-2× Age	11.60	<.0001		
							Bcl-2	12.39	<.0001		
−16	−81	−11	119	Left	Lingual Gyrus	Brodmann area 18	Age	33.68	<.0001	−0.50*	−0.07
							Bcl-2× Age	13.99	<.0001		
							Bcl-2	18.09	.009		
38	−59	13	60	Right	Middle Temporal Gyrus	Brodmann area 19	Age	11.09	<.0001	−0.32*	−0.04
							Bcl-2× Age	32.36	<.0001		
							Bcl-2	9.36	.002		
28	−15	−13	71	Right	Parahippocampal Gyrus	Hippocampus	Age	10.29	.001	−0.35*	−0.15
							Bcl-2× Age	11.06	<.0001		

Z-scores are for the peak statistically significant voxel for each regional cluster with uncorrected *P*≤.001 controlling for sex and education level.

−Indicated that there is no Brodmann area region around the center of a 5-mm radius search range.

* The *P* value of correlation between regional GMV and age less than.05; Abbreviations: MNI, Montreal Neurological Institute.

## Discussion

Our study represents the first investigation of Bcl-2 influences on age-related changes in brain morphology in healthy participants over a wide age range. The regional GM volumes of the right cerebellum, bilateral lingual gyrus, right middle temporal gyrus, and right parahippocampal gyrus were inversely correlated with age. However, the downward slope of the age-related reduction in GM was steeper in the A-allele carriers than in G homozygotes. Our findings support the hypothesis that Bcl-2 polymorphism may influence aging processes in the brain, and that the G/G allelic variant confers partial protection against age-related decreases in brain volume.

Many neuropathological studies have shown that normal aging is characterized by a substantial and extensive loss of neurons in the cerebral cortex. Morphometric imaging studies have demonstrated that aging predominantly affects the GM, including cortical and deep GM structures and the cerebellum [1], [35]. We found an accelerated loss in regional GM volumes with aging, which is consistent with the findings of previous studies [3], [35].

Bcl-2 has been shown to regulate neuronal cell death during normal development, and has also been implicated in many models of acute and chronic neurodegeneration [36]. Bcl-2 expression in the brain is up-regulated in Parkinson disease [37] and Alzheimer disease, with Bcl-2 expression increasing with increased disease severity [38]. The over-expression of Bcl-2 inhibits neuronal cell death in vitro [39], [40] and in vivo [41], [42]. Tanabe et al. [43] showed that endogenous Bcl-2 regulates neuronal cell survival in the central nervous system, and that Bcl-2 deficiency reduces neuronal viability under various adverse cellular conditions. Considering the anti-apoptotic properties of Bcl-2 in neurodegeneration, our findings support those of Machado-Vieira et al. [20], in which the Bcl-2 G/G genotype was associated with increased Bcl-2 mRNA and protein expression. Previous studies have observed that higher Bcl-2 expression may protect against dysfunctional calcium homeostasis in bipolar disorder patients [44]. Because Bcl-2 expression in the brain changes with age and increased expression of Bcl-2 may prevent or delay neuronal death [25], [42], [45], our findings suggest a potential genetic effect of Bcl-2 rs956572 in brain aging.

In our study, the protective effect of the homozygous Bcl-2-G allele was limited to the right cerebellum, the bilateral lingual gyrus, the right middle temporal gyrus, and the right parahippocampal gyrus. Thus, these regions may be sensitive to Bcl-2 modulation during brain aging. We observed that the cerebellum was most significantly affected by the Bcl-2 genotype. The Bcl-2 protein is widely expressed during the development of the nervous system, but is principally retained in specific regions of the brain, including the cerebellum [45]. Hochman et al. [46] found that Bcl-2-knockout mice displayed increased susceptibility to cellular oxidative processes and a loss of neurons in the cerebellum, which suggest that neuronal viability in the cerebellum may be influenced by Bcl-2. Kaufmann et al. [25] found that level of Bcl-2 expression was higher in the central nervous system of older rats, especially in the cerebellum, and increased oxidative stress has been observed in the cerebellum of aged animals [47]. If the increased expression of Bcl-2 represents a response to age-related oxidative challenge and cerebellum is highly susceptible to this challenge [25], the higher level of Bcl-2 expression from the homozygous G allele may protect against the age-related loss of neurons in the cerebellum.

Our study also demonstrated that Bcl-2 polymorphism influences the GM volume in the bilateral lingual gyrus, the right middle temporal gyrus, and the right parahippocampal gyrus. These findings are consistent with two previous imaging analyses of the genetic effects of Bcl-2. Salvadore et al. [23] reported that Bcl-2 rs956572 was associated with GM volume in the subcortical structures. Our prior study found that the Bcl-2 genotype could modulate GM volume in the lingual gyrus and middle temporal gyrus in elderly men [24]. The distribution of Bcl-2 varies among these regions, and the level of Bcl-2 expression has been shown to be associated with neurotoxin-triggered apoptosis and cellular injury [25], [45], [48], [49]. During the development of the human central nervous system, Bcl-2 expression declines gradually at more advanced stages, and an inverse correlation between apoptosis and Bcl-2 expression occurs in the areas surrounding the lingual gyrus [50]. Postmortem evidence supports apoptotic involvement in neuropsychiatric disorders, and low levels of Bcl-2 protein have been demonstrated in the middle temporal gyrus [51]. Furthermore, the hippocampus is particularly vulnerable to oxidative stress during aging, and altered Bcl-2 expression has been reported in the hippocampal region of aged rat [25]. Because the age-related changes in GM volume in these brain regions may be associated with Bcl-2 expression, differences in Bcl-2 expression levels among the Bcl-2 rs956572 allelic variants may influence the age-related rates of GM volume decline in these regions.

Based on our findings, the Bcl-2 rs956572 polymorphism has the most prominent effect on age-related GM volume reductions in the cerebellum. Significant interconnections of the cerebellum with the hippocampus and the occipital and temporal regions of the cerebral cortex have been implicated in the integration of sensory information, visuospatial organization, visual memory, procedural learning, and the control of behavior and motivation [52]–[56]. Because the cerebellum may have extensive outgoing connections to these regions, Bcl-2 rs956572 polymorphism may indirectly modulate GM volume reduction in the lingual gyrus, the middle temporal gyrus, and the parahippocampal gyrus through direct impacts on the cerebellum.

In our study, the age-related reduction in GM volume in the frontal and parietal lobes were not associated with Bcl-2 genotype. Although Bcl-2 expression is widespread in all brain regions, the effect of Bcl-2 expression on the trajectory of maturation or degeneration during brain aging may vary considerably in the cortex [50]. Analysis of post-mortem brain samples from patients with Alzheimer disease showed that the level of Bcl-2 expression were significantly higher in the cerebellum than in the frontal lobe [57]. Therefore, the effect of the Bcl-2 genotype on age- or neuropsychiatric disease-related changes in regional GM volumes warrants further investigation.

The need for statistically sufficient sample sizes in imaging studies of genetic variation has become increasingly recognized. The relatively large and, by international standards, homogenous sample of participants that were reviewed in our study lend credibility to our findings, based on previously proposed recommendations regarding cohort sizes [58]. However, the cross-sectional nature of our study design may represent a limitation to our findings. Prospective studies have demonstrated greater sensitivity for clarifying the GM volume changes in specific brain regions during the aging process [59]. In addition, it is possible that, rather than having a direct effect of GM volume, the Bcl-2 rs956572 polymorphism may be in linkage disequilibrium with the truly associated allele. Such linkage likely varies among different populations, which would confound the generalization of findings based on a homogenous Chinese cohort, such as ours. Furthermore, the addition of a clinical control group with a psychiatric disorder, such as bipolar disorder, to future study designs may yield added knowledge of the dual role of Bcl-2 in aging and disease states.

In conclusion, our findings of the effects of Bcl-2 rs956572 polymorphism on age-related morphologic changes in the brain indicate that Bcl-2 G homozygosity confers a protective effect against age-related GM volume reduction in several brain regions, particularly in the cerebellum. Although the underlying molecular mechanisms remain unclear, our findings support the hypothesis that Bcl-2-related genetic factors play a critical role in the effects of aging in the brain.
